# Supporting elimination of lymphatic filariasis in Samoa by predicting locations of residual infection using machine learning and geostatistics

**DOI:** 10.1038/s41598-020-77519-8

**Published:** 2020-11-25

**Authors:** Helen J. Mayfield, Hugh Sturrock, Benjamin F. Arnold, Ricardo Andrade-Pacheco, Therese Kearns, Patricia Graves, Take Naseri, Robert Thomsen, Katherine Gass, Colleen L. Lau

**Affiliations:** 1 Research School of Population Health, Australian National University, Canberra, Australia; 2 Global Health Group, University of California, San Francisco, San Francisco, USA; 3 Proctor Foundation, University of California, San Francisco, San Francisco, USA; 4 Menzies School of Health Research, Charles Darwin University, Brisbane, Australia; 5 College of Public Health, Medical and Veterinary Sciences, James Cook University, Cairns, QLD Australia; 6 Ministry of Health, Apia, Samoa; 7 Neglected Tropical Diseases Support Center, Task Force for Global Heath, Decatur, GA USA

**Keywords:** Health care, Public health, Epidemiology, Environmental impact, Infectious diseases

## Abstract

The global elimination of lymphatic filariasis (LF) is a major focus of the World Health Organization. One key challenge is locating residual infections that can perpetuate the transmission cycle. We show how a targeted sampling strategy using predictions from a geospatial model, combining random forests and geostatistics, can improve the sampling efficiency for identifying locations with high infection prevalence. Predictions were made based on the household locations of infected persons identified from previous surveys, and environmental variables relevant to mosquito density. Results show that targeting sampling using model predictions would have allowed 52% of infections to be identified by sampling just 17.7% of households. The odds ratio for identifying an infected individual in a household at a predicted high risk compared to a predicted low risk location was 10.2 (95% CI 4.2–22.8). This study provides evidence that a ‘one size fits all’ approach is unlikely to yield optimal results when making programmatic decisions based on model predictions. Instead, model assumptions and definitions should be tailored to each situation based on the objective of the surveillance program. When predictions are used in the context of the program objectives, they can result in a dramatic improvement in the efficiency of locating infected individuals.

## Introduction

Lymphatic filariasis (LF) is a globally widespread mosquito-borne disease caused by infection from one of three parasites, predominantly *Wuchereria bancrofti* (also *Brugia malayi* and *B. timori*)^1^*.* In severe cases, it can result in lymphedema, for which symptoms include elephantiasis (irreversible swelling of legs and thickening of the skin) and scrotal hydroceles in males, both of which have a major impact on quality of life. The World Health Organization (WHO) Global Programme to Eliminate LF (GPELF) was initiated in 2000, and as of 2018 has led to the treatment of 910 million people living in 68 countries^1^. GPELF has two major strategies for reducing the public health burden of LF: eliminating transmission, and managing morbidity of already affected persons. The major elimination strategy for breaking transmission is mass drug administration (MDA) to interrupt transmission in affected populations.

In Samoa, MDA has previously consisted of a two-drug combination (diethylcarbamazine citrate and albendazole), with the addition of a third drug (ivermectin) recently recommended for areas where LF transmission persists after multiple rounds of two-drug MDA^2^. Samoa has conducted eight nationwide rounds of MDA between 1998 and 2011^3^ and two subsequent subnational rounds in 2013 and 2017. Despite this effort, persistent LF remains, and in 2018 Samoa was the first country to distribute nationwide triple drug MDA using ivermectin, diethylcarbamazine citrate and albendazole (IDA)^3^.

A key challenge for LF elimination is that residual clusters of infection can persist even after the WHO-recommended number of rounds for population-wide MDA. Residual infection hotspots have been reported in Ghana after 11 rounds of MDA^4^, Sri Lanka after eight rounds^5^, American Samoa after seven rounds^6^, and Samoa after six rounds^7^. Clustering within households (households with higher than expected prevalence), as well as within neighbouring households has also been reported^7–10^. Locating these residual infections is imperative as it provides information on areas or subsections of the population where the program has not performed effectively and facilitates targeted treatment to help prevent resurgence of transmission.

The WHO recommended tool for detecting whether transmission is likely to have been interrupted following MDA is the Transmission Assessment Survey (TAS), typically conducted in 6–7 year old children through schools^11^. While 6–7 year olds have been selected as the most suitable age group for detecting recent infections, there is evidence that the TAS may have limited sensitivity for locating hotspots in some settings^5,6,10,12^. This may be in part due to its emphasis on providing overall prevalence estimates for an implementation unit, rather than focusing on identifying areas with expected high risk.

We propose that a targeted sampling strategy using predictions from a geospatial model trained on environmental variables and the location of known infections could be used to improve the efficiency of identifying the locations of infected people. By using this model to predict locations with a high risk of infection, sampling could potentially be targeted to increase the chances of finding residual infections. In this paper, we explored the use of geostatistics and machine learning (ML) to predict locations with a high risk of LF infection in Samoa, and focused on two research questions. Firstly, we examined whether a targeted sampling approach (sampling only households predicted by the model as being in a high-risk location) would be more effective at locating residual infections than random sampling. Secondly, we examined whether the effectiveness of this targeted sampling approach varied depending on the cut-offs used to classify locations as high or low risk based on the model predictions.

## Results

### Field survey and antigen prevalence

To evaluate the effectiveness of triple drug MDA (using IDA) in Samoa, a baseline survey of LF antigen prevalence was conducted in 2018 among residents aged ≥ 5 years, with a follow up survey in 2019, between six and nine months after the MDA. Household surveys across these two years carried out 4916 valid LF antigen tests from individuals aged 5 years or older. Based on household data, crude prevalence at the level of primary sampling unit (PSU) varied from 0–15.3% in 2018, to 0–18.7% in 2019 showing high between-PSU variability (median 4.4% in 2018, 2.6% in 2019; Table 1). Prevalence was higher in the five purposively selected villages compared to the 30 randomly selected villages (Supplementary Table S1).

**Table 1:** Study population and observed antigen prevalence from 2018 and 2019 household surveys.

Year	Number of households	Average household size	Number of participants	Crude antigen prevalence in household members (95% CI)	Median Crude antigen prevalence by PSU (range)
2018	495	4.8 (min 1, max 26)	2322	4.4% (3.7–5.4%)	4.4% (0–15.3%)
2019	542	4.8 (min 1, max 20)	2594	4.7% (4.0–5.6%)	2.6% (0–18.7%)

Sample size and crude antigen prevalence from household surveys across 35 primary sampling units in Samoa (participants aged ≥ 5 years).

### Model parameters and output

Rather than classifying locations as high or low risk based on predicted antigen prevalence, we classified locations using exceedance probabilities which represent the model’s posterior probability that prevalence is above a pre-determined threshold^13^. In the first instance, we used a prevalence threshold of 5% (based on the 4.4% average prevalence in 2018) and classified locations as predicted high risk (PHR) if the exceedance probability was > 70% (determined as the acceptable certainty threshold based on program objectives). By this definition, locations with at least a 70% probability that antigen prevalence was > 5% were classified as predicted high risk. All other locations were classed as predicted low risk (PLR). Of the 542 households surveyed in 2019, 10 (1.8%) were classified as being in a PHR location based on this definition. The effect of these cut-offs is examined in “Varying the definition of predicted high risk (PHR)”.

When the environmental covariate derived from the random forest model was excluded from the model, the Akaike information criterion (AIC) increased from 483 to 510, supporting the inclusion of environmental variables in the model. When we evaluated covariate importance in the random forest model using Gini impurity^14^, the median Normalized Difference Water Index (NDWI) was the most influential environmental variable, with elevation being the least influential (Table 2).

**Table 2:** Relative importance of environmental variables in the random forest model rated by Gini importance (mean decrease in Gini impurity); variables with high Gini importance values have a higher predictive value.

Variable	Gini importance
Median normalized difference water index	5.39
Distance to coast	5.06
Distance to water	4.99
Night time light intensity	4.15
Precipitation seasonality	3.45
Slope	3.27
Temperature seasonality	2.64
Annual mean temperature	2.62
Annual precipitation	2.48
Elevation	2.41

### Prevalence at predicted high risk locations

**Figure 1:**
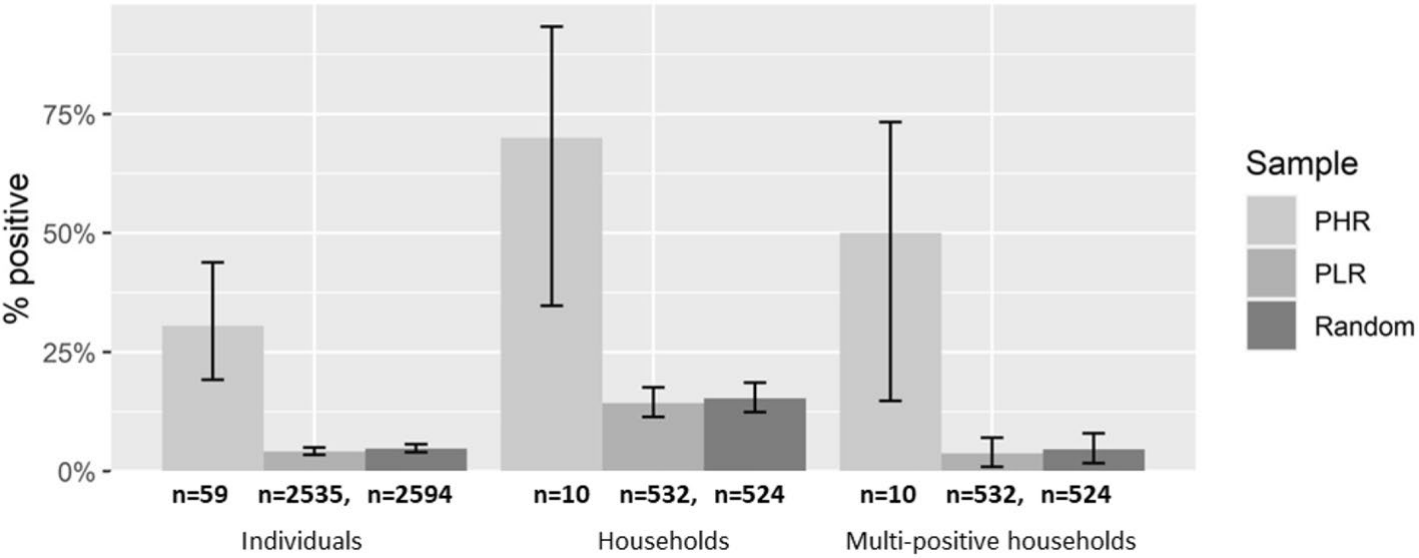
Proportion of individuals that tested positive (Individuals), proportion of households with at least one positive resident (Households) and proportion of households with two or more positive residents (Multi-positive households), stratified by PHR and PLR locations. The complete sample of all houses (random) is also shown. Values listed under each column indicate the total number of individuals or households in each category.

Both the proportion of antigen-positive individuals, and the proportion of households with at least one antigen-positive resident were significantly higher for the households at PHR locations compared to PLR locations, based on the two proportions z test (*p* < 0.05) (Fig. 1). This trend was also observed for multi-positive households (those with two or more positive residents).

The odds ratio for the presence of antigen-positive person(s) in PHR vs PLR locations was 10.2 (95% CI 4.2–22.8) for individuals, 14.0 (95% CI 3.64–53.8) for positive households and 25.6 (95% CI 7.69–85.2) for multi-positive households. Four of the 10 PHR household locations were in PSUs which had been purposively select for sampling because of previously high antigen prevalence. When the five purposive PSUs were removed from the testing sample (limiting predictions to only the 30 randomly selected PSUs), the odds ratio for identifying a positive household at PHR vs PLR locations was 8.0 (95% CIs 1.5–42.6), indicating that the model still successfully distinguished between high and low risk locations when tested on an unbiased, random sample.

### Varying the definition of “predicted high risk (PHR)”

**Figure 2:**
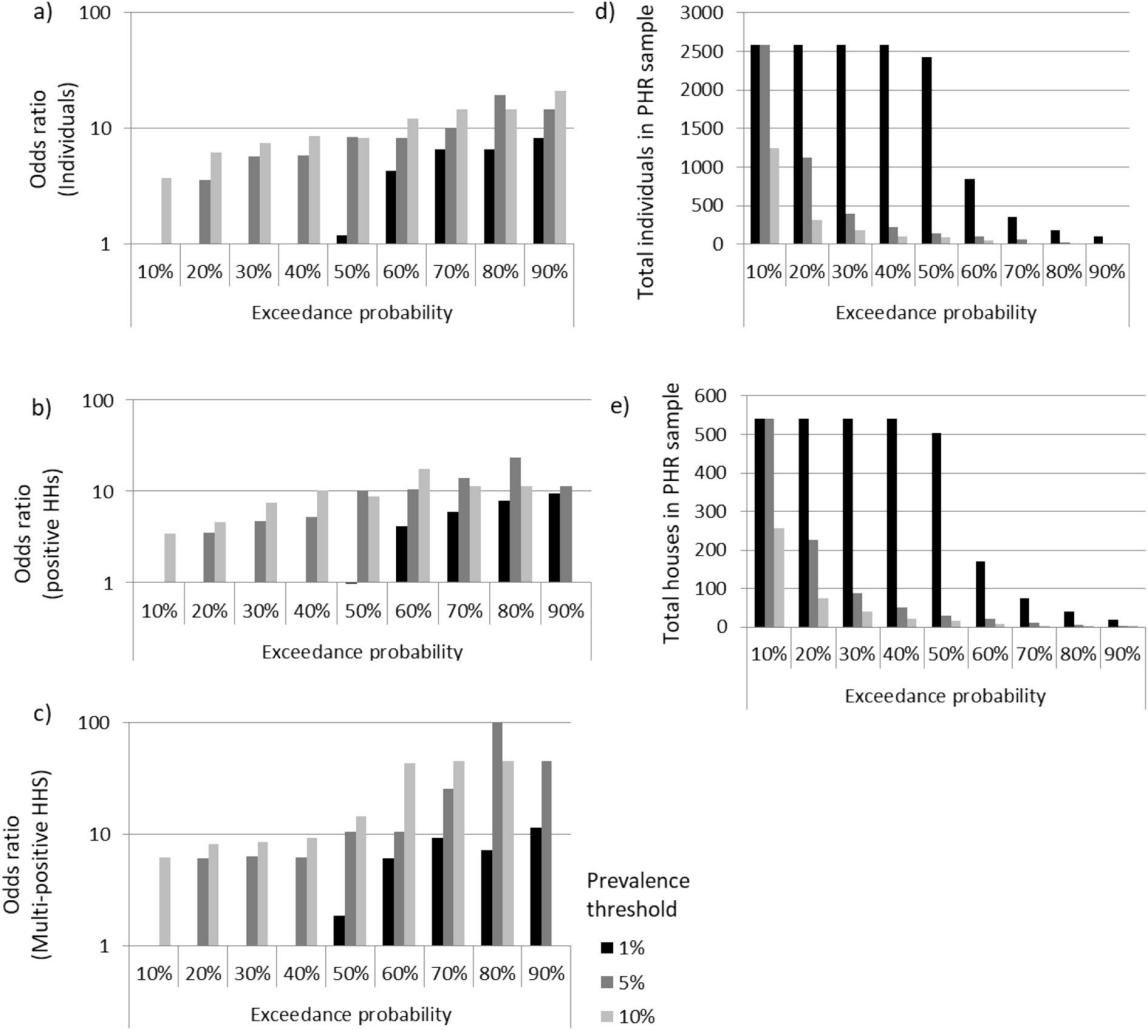
Odds ratios for (**a**) positive individuals, (**b**) positive households and (**c**) multi-positive households in predicted high risk compared to predicted low risk locations when stratified based on different combinations of prevalence thresholds (1%, 5%, 10%) and exceedance probabilities (10–90%). Values for predicted high risk definitions that had insufficient positive or multi-positive houses within either the predicted high risk or predicted low risk locations have been left blank. The number of (**d**) individuals and (**e**) households that were classified as being in a predicted location under each definition is also given.

The odds ratios for finding a positive individual, positive household or multi-positive household at PHR compared to PLR locations varied depending on the prevalence cut-offs and exceedance probability used to stratify the sample into PHR and PLR locations (Fig. 2 and Supplementary Table S2).

**Figure 3:**
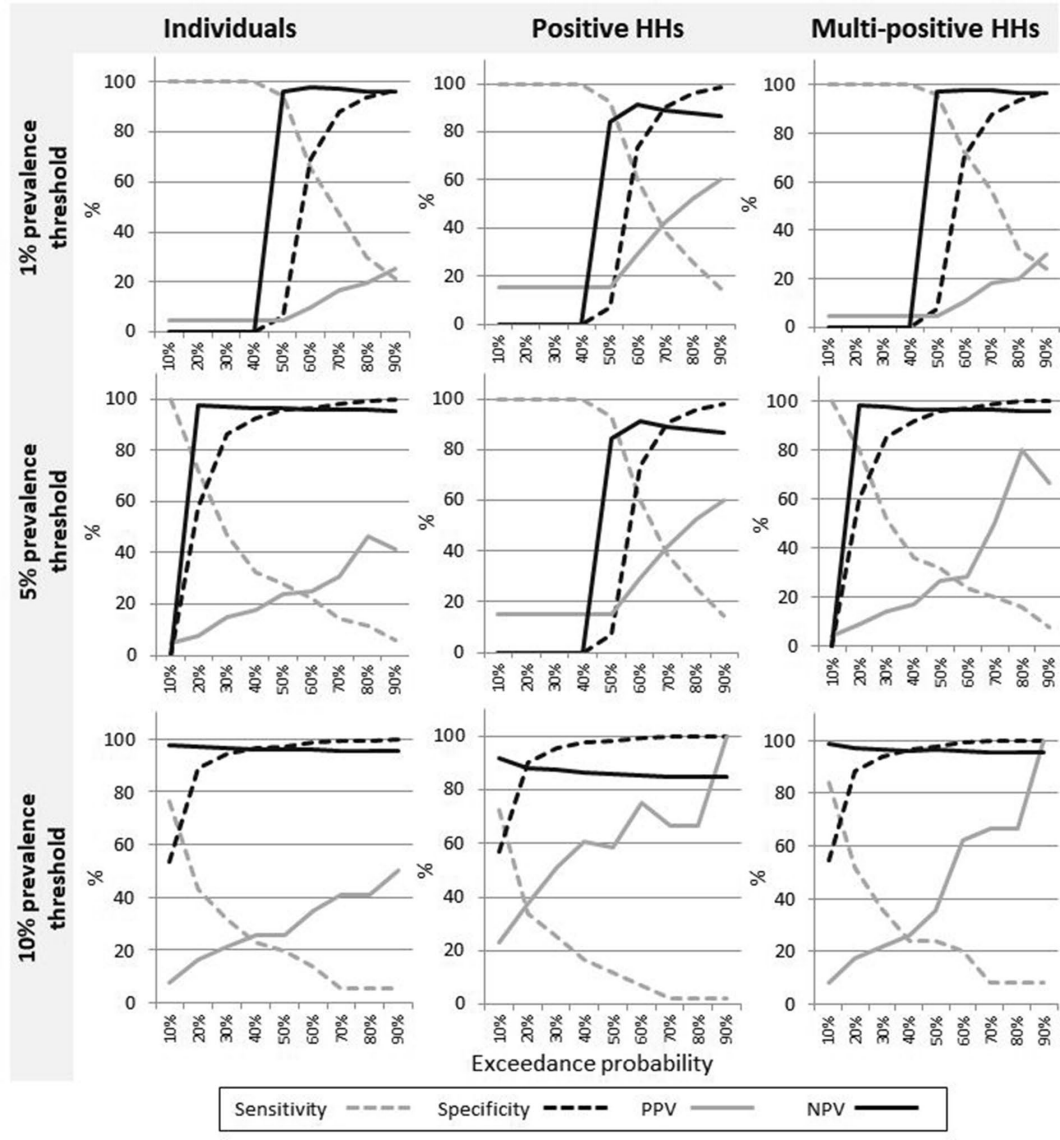
Model performance metrics with varying prevalence cut-offs and exceedance probability thresholds for defining predicted high risk locations. Sensitivity, specificity, positive predictive value, and negative predictive value are shown for individuals, positive households, and multi-positive households at prevalence cut-offs of 1%, 5% and 10%.

In general, using stricter definitions of high risk (increasing either the prevalence threshold or the exceedance probability) increased the odds for finding a positive person; however, for very strict or very lax definitions, there were insufficient locations in either of the PHR or PLR samples to be able to make a comparison. The confidence interval range also increased as either the prevalence threshold or exceedance probability was raised, reflecting a decrease in the number of houses defined as PHR (Supplementary Table S2). The sensitivity, specificity, positive predictive value (PPV) and negative predictive values (NPV) for positive individuals, positive households and multi-positive households within the PHR locations also varied substantially depending on the prevalence threshold and exceedance probability (Fig. 3).

Increasing the exceedance probability threshold tended to increase the model specificity (a reduction in false positives). In this context, this equates to fewer antigen-negative people being sampled for each antigen-positive person identified. However, this benefit was balanced by a decrease in sensitivity (more false negatives, representing an increase in the number of antigen-positive people who were not identified).

**Figure 4:**
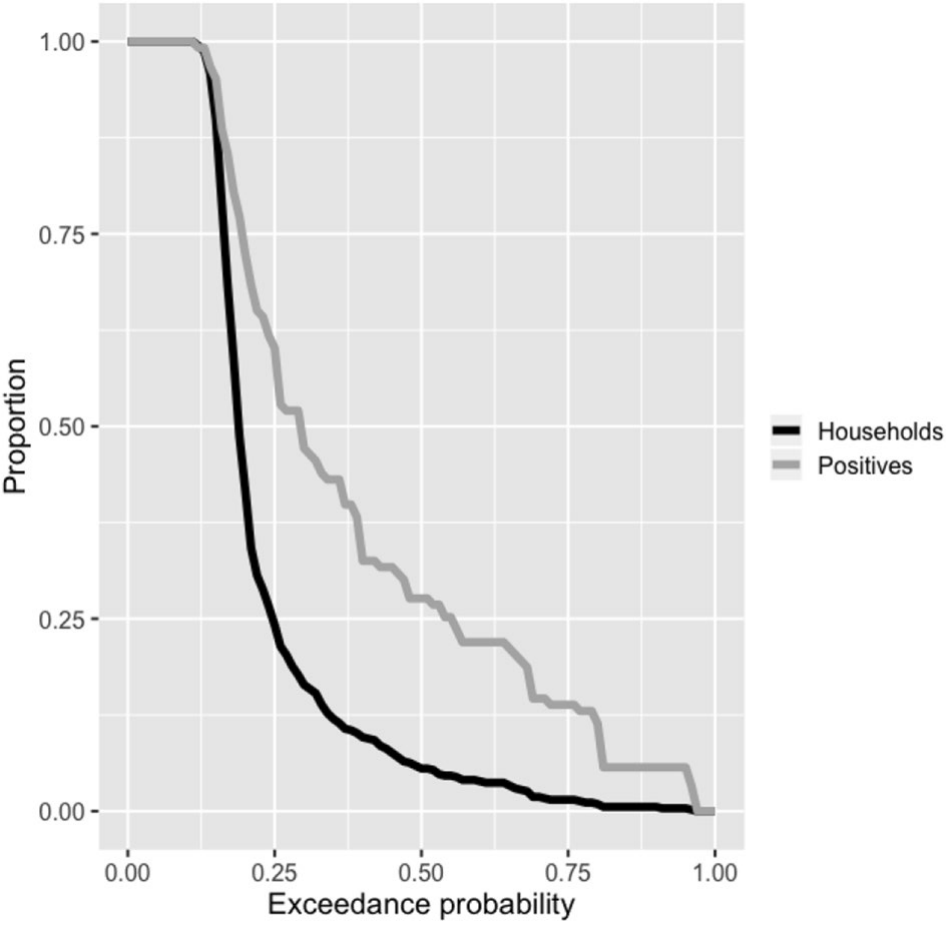
Proportion of houses sampled (black line) and proportion of positive individuals identified (grey line) if predicted high risk locations are sampled based on varying levels of exceedance probability used to define predicted high risk (for a 5% predicted prevalence cut-off).

A comparison of the proportion of houses sampled and proportion of positive individuals identified by exceedance probability (Fig. 4) shows that a targeted approach based on the model presented here is more efficient than random sampling. For example, using our model-driven approach, and an exceedance probability of 25%, it would be expected that only 17.7% of households would need to be sampled in order to find 52.0% of the positive individuals, meaning that only a small fraction of locations need to be sampled to find a larger fraction of infections.

## Discussion

Our study in Samoa showed that predictions from a spatial model can be used to guide a targeted sampling strategy that is more efficient than random sampling for locating residual LF infection at the household level. Model predictive performance varied depending on the cut-offs used to stratify locations into PHR and PLR. Stricter definitions (higher cut-offs for either the prevalence threshold or exceedance probability) resulted in an increase in PPV, but also lowered sensitivity. For practical purposes, in a disease elimination scenario where on the ground resources are finite and generally insufficient for exhaustive sampling, this represents a trade-off between the risk of surveying a household with no infected residents and the risk of not surveying a household with infected residents (i.e. between the number of houses sampled and the number of infections found). In a decision-making context, model predictions can be used to prioritise which houses to sample based on the available resources in order to maximise the number of infected people who are located and offered treatment.

There are at least two real world scenarios where these results are applicable. In a low prevalence setting, such as in an end-game scenario and the tail-end of an elimination program, decision makers may decide to do focal MDA in any village where the prevalence is thought to exceed a certain threshold (e.g. 1% as for TAS). In that case, a more cautious definition of high risk (i.e. lower thresholds) could be applied to maximize the finding of positive cases to give an early warning as to when an MDA may be required. Alternatively, where survey resources are limited or testing procedures are invasive, the objective becomes to locate and treat as many infections as possible, while testing as few non-infected persons as possible. In this scenario a higher prevalence threshold would be more appropriate, combined with a targeted sampling approach that prioritizes locations with higher exceedance probabilities (certainty). As Fig. 4 shows, this strategy could prove efficient.

The use of data-driven models based on fine scale geo-referenced data creates the potential to utilize the results of existing monitoring and surveillance programs to direct future surveys. In this study, data from 2018 were used to predict high risk locations the following year, however future tools based on this technology (such as online tools) would allow for a faster turn-around as part of ongoing monitoring and surveillance programs, or as aids to locating infections as part of a targeted treatment program. For example, initial results from random surveys could be analyzed using the model and the results used to then strategically target predicted high risk locations. The results presented here indicated that this strategy could potentially result in a higher number of cases being treated than if simple random sampling had been used. The model does however rely on having recent infection data relative to the disease in question, so is designed to be used as a complement to existing monitoring programs.

A key strength of this study is the high spatial accuracy of the data. Capturing household coordinates using both GPS on smart phones as well as on paper field maps allowed for locations for the majority of households to be recorded at an accuracy of less than 30 m. While inevitably a small proportion of houses may have less accurate locations, the large sample size means that the effect of any misidentified locations will have negligible effect on the overall results.

While environmental variables, particularly Normalized Difference Water Index and distance to water, improved model predictions, results were strongly influenced by the proximity to households with known infections, as indicated by the small increase in AIC when the environmental variables were not considered. This is consistent with previous findings of LF in other settings^15^ and suggests the presence of smaller scale factors not included in the model. The environmental predictors used for training our model were based on data availability and previous work on predicting hotspot villages for malaria^16,17^. While they are still potentially suitable for other vector-borne diseases, such as LF, additional environmental or demographic factors that have been specifically shown as relevant predictors for LF, such as population density^15^, could potentially improve model predictions provided there is sufficient variation at the spatial scale at which predictions are being made. This would also be an important step towards models that are able to make useful predictions in villages that have not been previously sampled.

There are several factors that will determine whether or not a targeted sampling approach based on data modeling will provide a net benefit once the effort and resources required to run the model are considered. First, collecting the required geo-referenced data and running the model are resource intensive. This overhead can be reduced in situations where data which are already being collected as part of a wider monitoring program can be used as inputs (such as the data used in this study), and by the development of user-friendly interfaces, such as those currently being developed by The Disease Surveillance And Risk Monitoring project (DiSARM—www.disarm.io) and Reveal (www.revealprecision.com). Second, the infection prevalence within the community being sampled will impact the effectiveness of purely random sampling, and therefore the net benefit gained from any alternative. Our study is a first step towards generating evidence about the potential for using spatial models to inform sampling strategy. Further investigations are required to explore whether the gains from a model-driven targeted sampling approach are maintained when compared to other approaches such as adaptive cluster sampling^18–20^, and under what conditions. The lower odds ratio results for predictions made only on the randomly sampled PSUs indicate that the model may perform differently in lower prevalence settings. Further work is needed to examine this.

The data used in the study were collected for the evaluation of the impact of mass drug administration for the elimination of LF, which is being conducted as part of the Global Programme to Eliminate LF, and as such do not represent a truly random sample due to the five purposively selected PSUs. However, whereas randomly sampled data are required for unbiased estimates of population prevalence, population-representative samples are less important if the objective is to distinguish high risk from low risk locations. We therefore elected to train the model with the complete dataset, rather than the subset of the random 30 PSUs. By parameterizing the model with all available data, this ensured that the training data included sufficient samples from the full range of scenarios (high and low prevalence). The reported comparison shows that the model still performed well in the subsample of 30 randomly sampled PSUs.

As programs approach LF elimination, the number of infected individuals decreases and the location of these individuals becomes increasingly focal. Consequently, more effective sampling strategies that exploit the spatial relationships between positive individuals, as well as correlation with environmental indicators, can help programs to make more efficient use of limited resources. This study provides evidence that a ‘one size fits all’ approach is unlikely to yield optimal results when making programmatic decisions based on such model predictions. Instead, model assumptions and definitions should be tailored to each situation based on the best available information and the objective of the surveillance program. When predictions are used appropriately, and in the context of the program objectives, the algorithm can result in a dramatic improvement in the efficiency of finding locations with infected individuals.

## Methods

### Study region

Samoa, located in the South Pacific, has a population of approximately 200,000 people^21^. Ninety percent of the population live on the main islands of Upolu and Savai’i in a predominately rural or semi-rural setting. The main vector for LF in Samoa is *Aedes polynesiensis*, a day-biting mosquito that is highly efficient at disease transmission^22^.

### Field survey design

**Figure 5:**
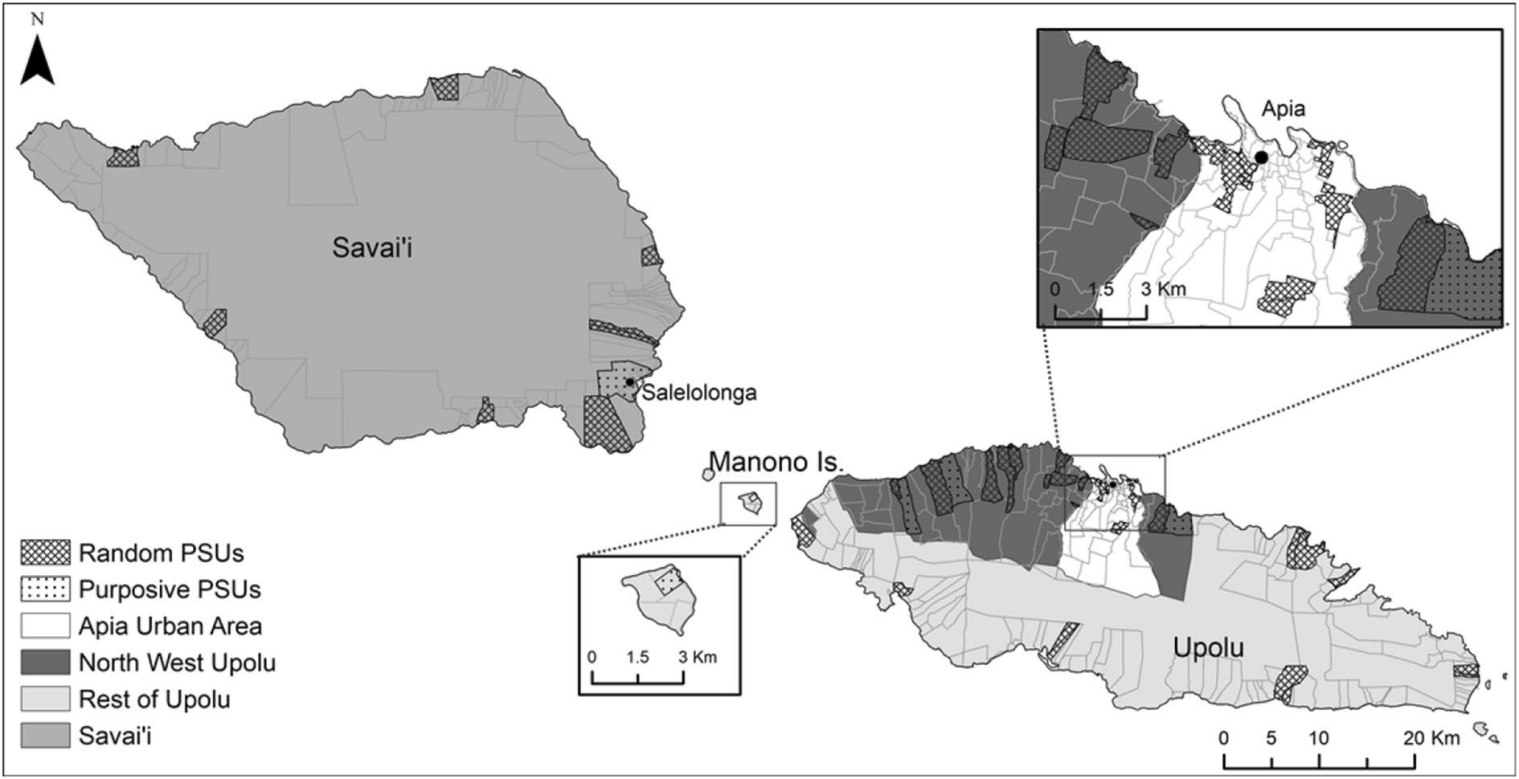
Location of random and purposive sampled primary sampling units in Samoa for 2018 and 2019 field surveys. Spatial data on country, island, region, and village boundaries in Samoa were obtained from the Pacific Data Hub (pacificdata.org) and DIVA-GIS (diva-gis.org). Geographic information systems (GIS) software (ArcGIS v10.4.1, Environmental Systems Research Institute, Redlands CA—www.esri.com) was used to produce maps.

This study used data from field surveys conducted in 2018 and 2019 as part of the Surveillance and Monitoring and Elimination of Lymphatic Filariasis and Scabies in Samoa project. The 2018^23^ survey was conducted within 3 months of the first round of IDA and the 2019 survey was conducted 6–9 months after the first round of IDA. In both surveys, the same 35 PSUs were surveyed, each consisting of one or two villages. Thirty of the 35 PSUs were randomly selected, and five were purposefully selected by the Samoan Ministry of Health due to high antigen prevalence in previous surveys (Fig. 5). The houses sampled within each PSU differed between years, although there was some overlap by chance, especially in the smaller villages.

The target sample size for each survey was calculated as the minimum proportion of the overall population required to detect a 1% prevalence threshold with an upper 1-sided confidence limit of 5% and 75% power. This equated to 104 participants per PSU each year (57 aged 5–9 years, and 57 aged ≥ 10 years). In each PSU, two sampling strategies were used to reach this sample size, with a convenience survey of 5–9 year old children being conducted concurrently with a household survey of those aged ≥ 5 years in approximately 15 households. Results of the convenience survey were not included in this study, as household locations for these participants were not recorded.

Households chosen for sampling were selected with a ‘virtual walk’ method, using GoogleEarth satellite image of the village and sequentially numbering every building that resembled a house. A random house was allocated as the initial starting point and the remaining 14 houses were selected at equal intervals from this location. Every selected house was visited as least once, and all eligible residents home at the time who agreed to participate were enrolled in the study. Buildings that were selected for sampling based on the virtual walk, but which were not domestic residences were replaced with either the nearest house or the house of the family who owned the building. Households with no-one home were revisited later in the day or the next day. If all residents declined to participate or were not home on the second visit, the house was replaced with the nearest household. Empty houses were only replaced if sufficient numbers of adults had not been sampled to meet the targets. Within a PSU, if the required sample size for participants aged ten years and over was not achieved after sampling 15 houses, a further five houses were randomly selected for sampling.

House locations were recorded using smartphones aiming for an accuracy of < 30 m, as well as marked manually on fieldwork maps. Final house locations were then consolidated from these two sources. GPS coordinates were used when the accuracy was 30 m or less and the location was within the boundary of the correct PSU. In other cases, the location was taken from the annotated field maps marked by the survey teams. At each household, finger prick blood samples (approximately 400 μL) were taken from each consenting resident aged five years or older. Informed consent was obtained from all subjects and for subjects under 18, from a parent and/or legal guardian. Samples were refrigerated until the next working day (maximum 48 h), after which filariasis test strips (FTS; www.alere.com) were used to detect the presence of LF antigens as described in^12^.

Ethical approval was obtained from human research ethics committees at the Samoa Ministry of Health and The Australian National University (protocol 2018/341). The study was conducted in collaboration with the Samoa Department of Health, WHO Samoa country office, Samoa Red Cross, The Task Force for Global Health, and the United States Centers for Disease Control and Prevention. permission was sought from village chiefs before entering a village and verbal and written consent was obtained from all participants, or from the parents or guardians of participants under the age of 18. All methods were performed in accordance with the relevant guidelines and regulations.

### Random forest-model based geostatistics ensemble methods

To model the relationship between environmental covariates and antigen positivity, we used a two-step process similar to that described in^24^ and^25^. Model predictions were made based on the house locations of infected persons identified from previous surveys, as well as environmental variables relevant to mosquito density, such as elevation and rainfall^26,27^. We chose to leverage a machine learning step to reduce the environmental covariates to a single dimension rather than including the covariates as linear terms in the geostatistical model, as this allows for complex interactions and non-linear effects, and previous studies using similar approaches show this reduces prediction error^24^.

Using the 2018 survey data, a binomial random forest was fit using the environmental variables. A random forest is a machine learning algorithm consisting of an ensemble, or forest, of decision trees and the technique is widely used for its predictive performance for classification and regression problems^28–31^. The environmental variables included in this model were elevation^32^, distance to inland water^33^, slope, VIIRS night time light intensity for 2016, distance to coastline^34^, mean annual temperature, temperature seasonality, annual precipitation, seasonality of precipitation^35^ and the Landsat median Normalized Difference Water Index calculated using Google Earth Engine across 2016–2018^36^. All covariates were resampled to 1 km resolution.

The influence of each of the environmental variables (covariates) was estimated using Gini importance (the mean decrease in Gini impurity)^37^. Gini importance describes how important a variable is for classifying locations as either predicted high risk or predicted low risk^14^. In addition to predicting probability of infection at every household (from here on termed in-sample predictions), tenfold cross validation was performed and cross-validated predictions at every sampled household location (from here on termed out-of-sample predictions) was retained.

Second, a geostatistical model was then fit (also using the 2018 data) to the infection data using out-of-sample predictions from the random forest as a single covariate. A spatially structured household level random effect was included, where spatial correlation was modelled using a Matérn correlation function^38^. Models were fit in R 3.5 using the ranger^39^ and spaMM packages^40^. The geospatial model was run with and without the environmental covariate and evaluated based on AIC.

To make predictions from the geostatistical model, the in-sample predictions from the random forest were used as a covariate. Rather than only predicting probability of infection at each household, a posterior distribution of probabilities was generated by conditionally simulating from the geostatistical model. In turn, these posterior distributions were used to estimate ‘exceedance probabilities’ or the probability that infection prevalence at any household location exceeds a given threshold.

The model stratifies locations into predicted high risk (PHR) or predicted low risk (PLR) based on a predicted probability determined by two parameters: (1) an antigen prevalence threshold of interest (likelihood that a person sampled at that location would be antigen positive), and (2) the exceedance probability, defined as the probability that prevalence at that location exceeds the threshold. This approach explicitly allows us to take advantage of the uncertainty estimates around predictions as opposed to relying solely on the best guess prevalence estimate. PHR house locations were defined as locations with at least a 70% chance of having a prevalence of 5% or higher. The 70% certainty threshold was chosen as an acceptable level of certainty for decision makers for identifying locations with above average risk of infection. The 5% seroprevalence cut-off was chosen to reflect the 4.4% prevalence from the 2018 survey.

#### Comparing antigen prevalence between PHR and PLR locations in 2019

We compared the PHR and PLR locations by evaluating (1) the proportion of individuals who tested positive (antigen prevalence), (2) the proportion of locations with at least one positive person (positive locations), and (3) the proportion of locations where more than one person tested positive (multi-positive locations). To calculate odds ratios and 95% confidence intervals, we used logistic regression with Huber-White robust standard errors to account for clustering at the PSU level^41^. Generalised linear models (GLMs) were run using the base stats package in R^42^ and Huber-White robust standard errors were calculated using the Sandwich package^43^. Odds ratios were calculated for positive individuals, positive households, and multi-positive households at PHR locations compared to PLR locations. This was calculated firstly for the entire 2019 household sample, and secondly based only on results from the 30 randomly (as opposed to purposively) sampled PSUs.

#### Classification cut-offs used to define PHR locations

We conducted further analyses to explore changes in model performance using different combinations of antigen prevalence threshold and exceedance probability. To evaluate the extent to which changing the definition of ‘predicted high risk’ affected the results, we calculated the log odds of locating a positive individual, positive household or multi-positive household in PHR compared to PLR locations under different definitions. Definitions were adjusted based on both the prevalence threshold (1%, 5%, 10%) and exceedance probability (10–90%). Each of these combinations were also assessed according to sensitivity, specificity, positive predictive value and negative predictive value^44^.

## Supplementary information

Supplementary Information

Supplementary Information

## Data Availability

All relevant data are within the paper. We are unable to provide individual-level antigen prevalence data and demographic data because of the potential for breaching participant confidentiality. The communities in Samoa are very small, and individual-level data such as age, sex, and village of residence could potentially be used to identify specific persons. For researchers who meet the criteria for access to confidential data, the data are available on request from the Human Ethics Officer at the Australian National University Human Research Ethics Committee, email: human.ethics.officer@anu.edu.au.
