# Supplementary material for: Supporting elimination of lymphatic filariasis in Samoa by predicting locations of residual infection using machine learning and geostatistics
Source: Sci Rep. 2020 Nov 25;10:20570. doi: 10.1038/s41598-020-77519-8 (PMC7689447; doi:10.1038/s41598-020-77519-8)
Supplement: Supplementary file 1 — Supplementary Information [file 41598_2020_77519_MOESM1_ESM.docx]

**Lymphatic filariasis elimination in Samoa: Predicting locations of residual infection using geostatistics and machine learning**

Helen J. Mayfield, Hugh Sturrock, Benjamin F. Arnold, Ricardo Andrade-Pacheco , Therese Kearns, Patricia Graves, Take Naseri, Robert Thomsen, Katherine Gass, Colleen L. Lau

# Supplementary

**Table S1:** Study population and observed antigen prevalence from 2018 and 2019 household surveys. Sample size and crude antigen prevalence from household surveys across 35 primary sampling units in Samoa (participants aged ≥5 years).

| Year and sample | Number of households | Average household size | Number of participants | Crude antigen prevalence in household members (95% CI) | Median  Crude antigen prevalence by PSU (range) |
| --- | --- | --- | --- | --- | --- |
| 2018 all PSUs | 495 | 4.8  (min 1, max 26) | 2322 | 4.4%  (3.7% - 5.4%) | 4.4%  (0% - 15.3%) |
| 2019 all PSUs | 542 | 4.8  (min 1, max 20) | 2594 | 4.7%  (4.0% - 5.6%) | 2.6%  (0% - 18.7%) |
| 2018 random PSUs | 433 | 4.6  (min 1, max 26) | 2008 | 3.7%  (3.0% - 4.7%) | 2.9%  (0% - 14.0%) |
| 2019 random PSUs | 467 | 4.7  (min 1, max 20) | 2182 | 3.4%  (2.7% - 4.3%) | 1.5%  (0% - 18.7%) |
| 2018 Purposive PSUs | 62 | 5.1  (min 1, max 13) | 314 | 8.9%  (6.0% - 12.6%) | 7.5%  (4.7% - 15.23) |
| 2019 Purposive PSUs | 75 | 5.5  (min 1, max 17) | 412 | 11.7%  (8.7% - 15.1%) | 12.7%  (4.5% - 16.0%) |
