# Supplementary material for: Supporting elimination of lymphatic filariasis in Samoa by predicting locations of residual infection using machine learning and geostatistics
Source: Sci Rep. 2020 Nov 25;10:20570. doi: 10.1038/s41598-020-77519-8 (PMC7689447; doi:10.1038/s41598-020-77519-8)
Supplement: Supplementary file 2 — Supplementary Information [file 41598_2020_77519_MOESM2_ESM.docx]

**Lymphatic filariasis elimination in Samoa: Predicting locations of residual infection using geostatistics and machine learning**

Helen J. Mayfield, Hugh Sturrock, Benjamin F. Arnold, Ricardo Andrade-Pacheco , Therese Kearns, Patricia Graves, Take Naseri, Robert Thomsen, Katherine Gass, Colleen L. Lau

# Supplementary

Table S2: Odds ratios for individuals, positive houses and multi-positive houses in predicted high risk compared to predicted low risk locations. The number of PHR house locations under each definition is also given.

| Prevalence cut-off | Exceedance Probability | OR - Individuals (95% CI) | PHR individuals | OR - Positive HHs (95% CI) | OR - Multi Positive HHs (95% CI) | PHR HHs |
| --- | --- | --- | --- | --- | --- | --- |
| 1 | 10 | - | 2594 | - | - | 542 |
|  | 20 | - | 2594 | - | - | 542 |
|  | 30 | - | 2594 | - | - | 542 |
|  | 40 | - | 2594 | - | - | 542 |
|  | 50 | 1.2 (0.5 - 2.9) | 2422 | 1 (0.4 - 2.5) | 1.9 (0.3 - 13.3) | 504 |
|  | 60 | 4.3 (2.3 - 7.9) | 846 | 4.2 (2.2 - 7.8) | 6.1 (2.7 - 13.8) | 172 |
|  | 70 | 6.6 (3.9 - 11.4) | 352 | 5.9 (3 - 11.7) | 9.3 (4.1 - 21.1) | 76 |
|  | 80 | 6.6 (3.7 - 11.7) | 188 | 7.8 (4.3 - 14.4) | 7.1 (3.1 - 16.6) | 40 |
|  | 90 | 8.3 (4.4 - 15.6) | 103 | 9.5 (4.2 - 21.8) | 11.4 (4.4 - 29.2) | 20 |
| 5 | 10 | - | 2594 | - | - | 542 |
|  | 20 | 3.6 (1.9 - 6.7) | 1131 | 3.5 (1.9 - 6.6) | 6 (2.4 - 15.2) | 226 |
|  | 30 | 5.8 (3.3 - 10.2) | 390 | 4.8 (2.6 - 8.8) | 6.3 (3 - 13.2) | 89 |
|  | 40 | 5.9 (3.2 - 10.7) | 227 | 5.2 (2.7 - 9.7) | 6.2 (2.4 - 15.9) | 52 |
|  | 50 | 8.4 (4.8 - 14.9) | 141 | 10.3 (4.9 - 21.6) | 10.6 (4.3 - 26.1) | 30 |
|  | 60 | 8.3 (4.5 - 15.2) | 108 | 10.5 (4.9 - 22.4) | 10.6 (4.3 - 26) | 21 |
|  | 70 | 10.2 (4.5 - 22.8) | 59 | 14 (3.6 - 53.8) | 25.6 (7.7 - 85.2) | 10 |
|  | 80 | 19.7 (8.5 - 45.5) | 30 | 23.2 (3.4 - 159.1) | 98.3 (13.3 - 727) | 5 |
|  | 90 | 14.9 (5.4 - 40.8) | 17 | - | - | 3 |
| 10 | 10 | 3.7 (2 - 7.2) | 1243 | 3.5 (1.8 - 6.6) | 6.3 (2.4 - 16.7) | 257 |
|  | 20 | 6.2 (3.6 - 10.7) | 321 | 4.6 (2.3 - 9.3) | 8.1 (3.9 - 16.8) | 74 |
|  | 30 | 7.5 (4.3 - 13) | 183 | 7.4 (4 - 13.9) | 8.5 (3.3 - 22.1) | 41 |
|  | 40 | 8.6 (4.8 - 15.3) | 110 | 10.1 (4.7 - 22.1) | 9.3 (3.7 - 23.5) | 23 |
|  | 50 | 8.3 (4.1 - 17.1) | 94 | 8.9 (3.7 - 21) | 14.5 (5.8 - 36.4) | 17 |
|  | 60 | 12.2 (5.3 - 28.4) | 49 | 17.8 (3.7 - 86.7) | 42.8 (12.6 - 146.1) | 8 |
|  | 70 | 14.9 (5.4 - 40.8) | 17 | - | - | 3 |
|  | 80 | 14.9 (5.4 - 40.8) | 17 | - | - | 3 |
|  | 90 | 21.2 (8.7 - 52.1) | 14 | - | - | 0 |
